# Supplementary material for: Slow Interstitial Fluid Flow Activates TGF-β Signaling and Drives Fibrotic Responses in Human Tenon Fibroblasts
Source: Cells. 2023 Sep 4;12(17):2205. doi: 10.3390/cells12172205 (PMC10486805; doi:10.3390/cells12172205)
Supplement: Supplementary file 1 [file cells-12-02205-s001.zip › cells-2475058-supplementary.pdf]

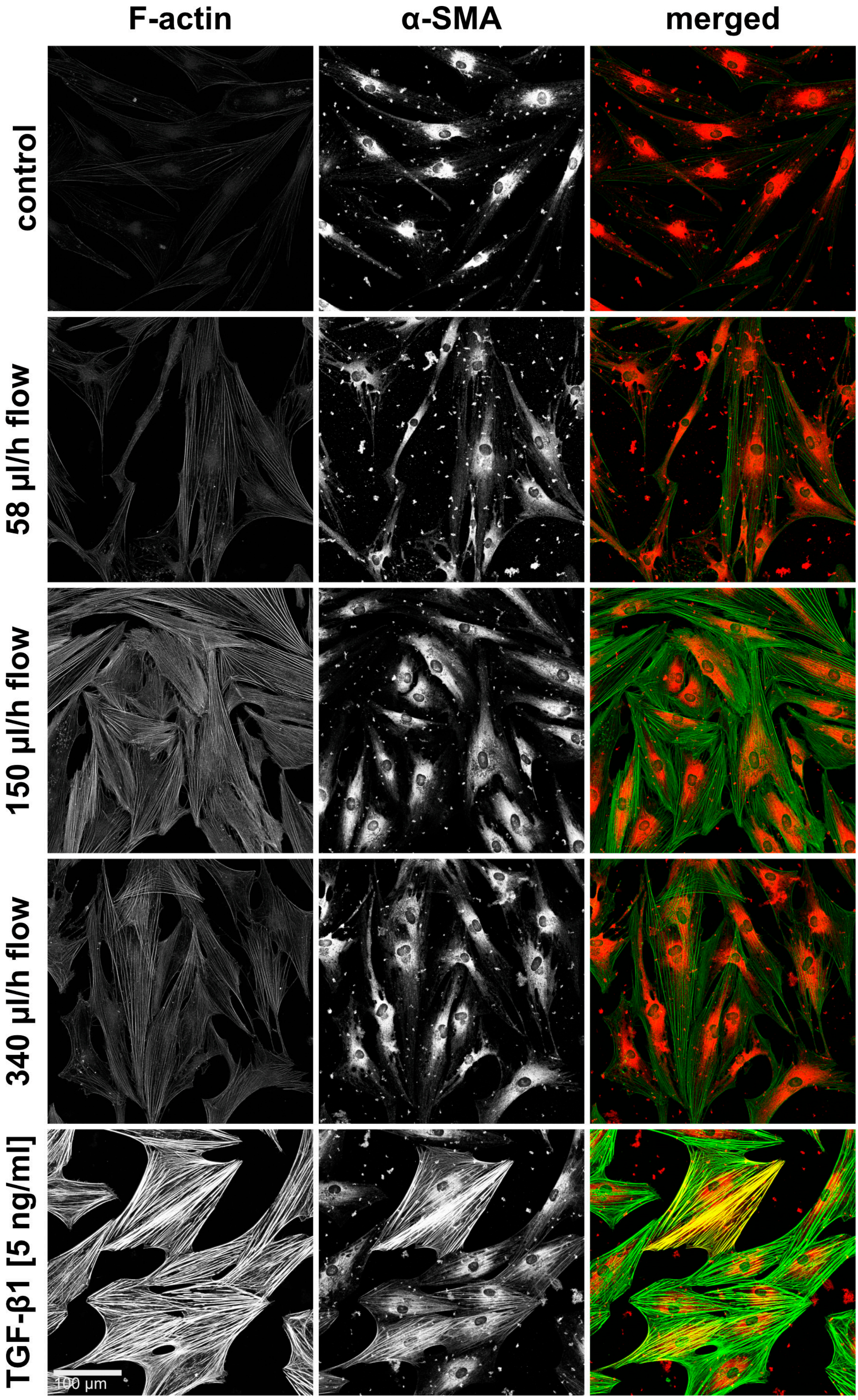
F-actin
α-SMA
merged
control
58 µl/h flow
150 µl/h flow
340 µl/h flow
TGF-β1 [5 ng/ml]
100 µm

Fig. S1: IF of F-actin and α-SMA under varying flow rates in the 2D model

Confocal immunofluorescence microscopy of F-actin and α-SMA in HTFs exposed to different flow rates or TGF--β1 (5 ng/ml). Cells were preincubated in μ-slides for 24 h in 0.2% FBS medium, then perfused for 72 h with the respective flow rate or stimulated with TGF--β1 (5 ng/ml) with 0.2% FBS medium. The result was representative of three independent experiments.

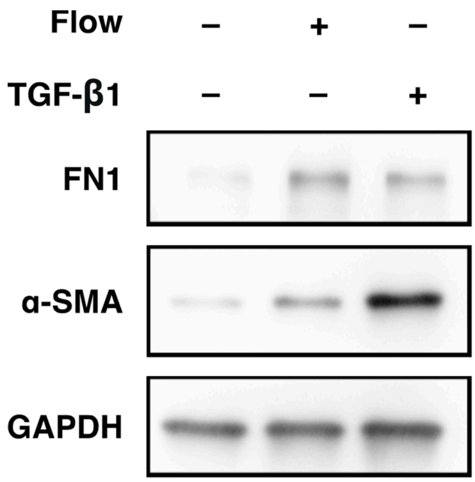

Fig. S2: Western blot of whole cell lysates under static conditions, flow conditions and TGF--β1 stimulation in the 3D model

Western blot analysis of protein levels in whole cell lysates after 72 hours is shown. In cells exposed to flow the flow rate was 666 μl/h. The TGF-β1-concentration was 5 ng/ml. GAPDH served as loading control. The result was representative of three independent experiments.

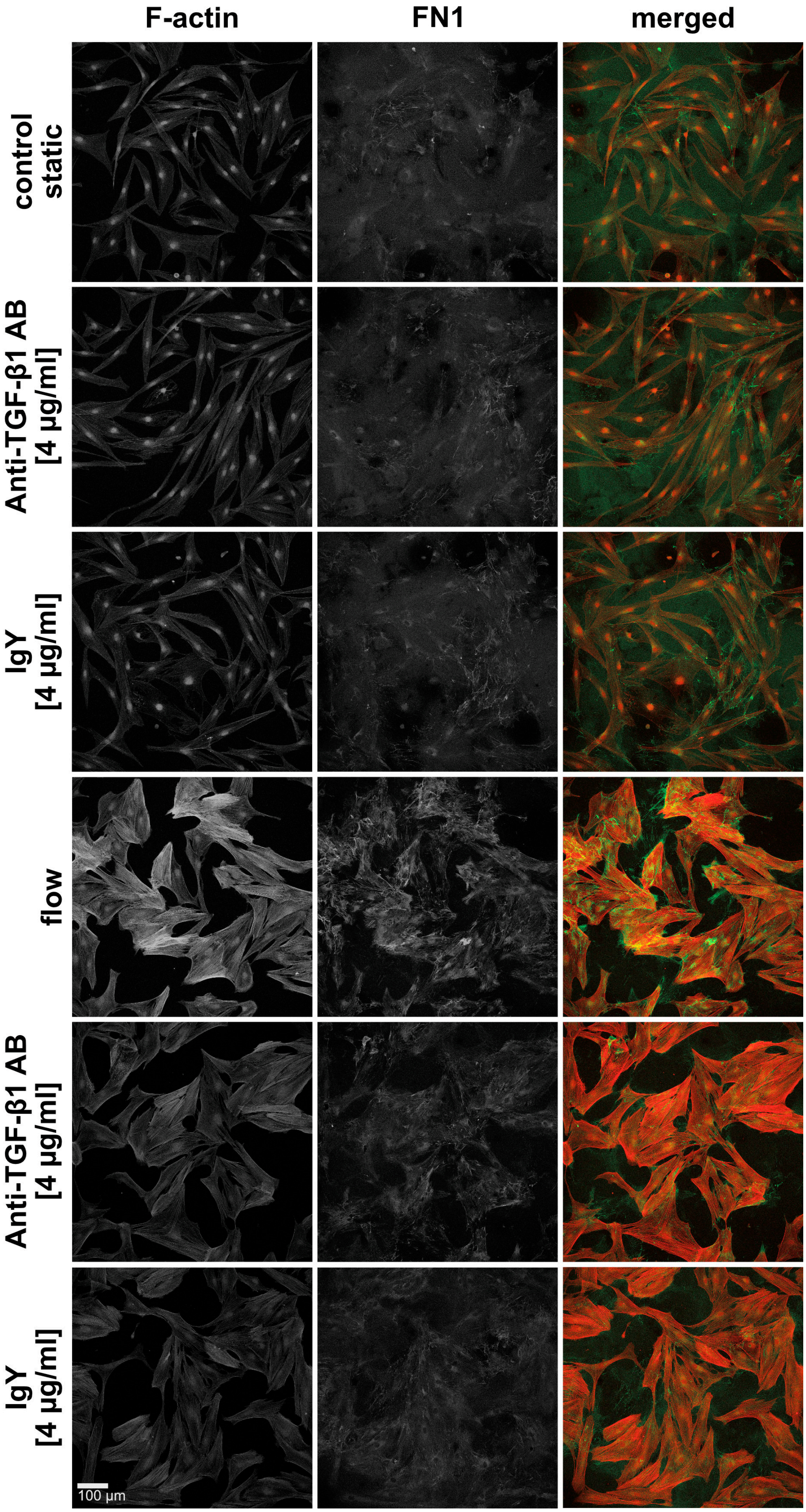

F-actin
FN1
merged
control static
Anti-TGF-β1 AB [4 µg/ml]
IgY [4 µg/ml]
flow
flow Anti-TGF-β1 AB [4 µg/ml]
flow IgY [4 µg/ml]
100 µm

Fig. S3: IF of F-actin and FN1 under static conditions and flow conditions with anti-TGF-β1-antibodies and IgY isotype controls in the 2D model

Confocal immunofluorescence microscopy of F-actin and FN1 in HTF with anti-TGF--β1 antibodies [4 µg/ml] and IgY isotype controls [4 µg/ml] under static and flow conditions (150 µl/h) in the 2 D model. Cells were preincubated in µ-slides for 24 h in 0.2% FBS medium, then incubated under static or flow conditions for 72 h. Image acquisition and representation settings are identical for all conditions. The figure is representative of three independent experiments.

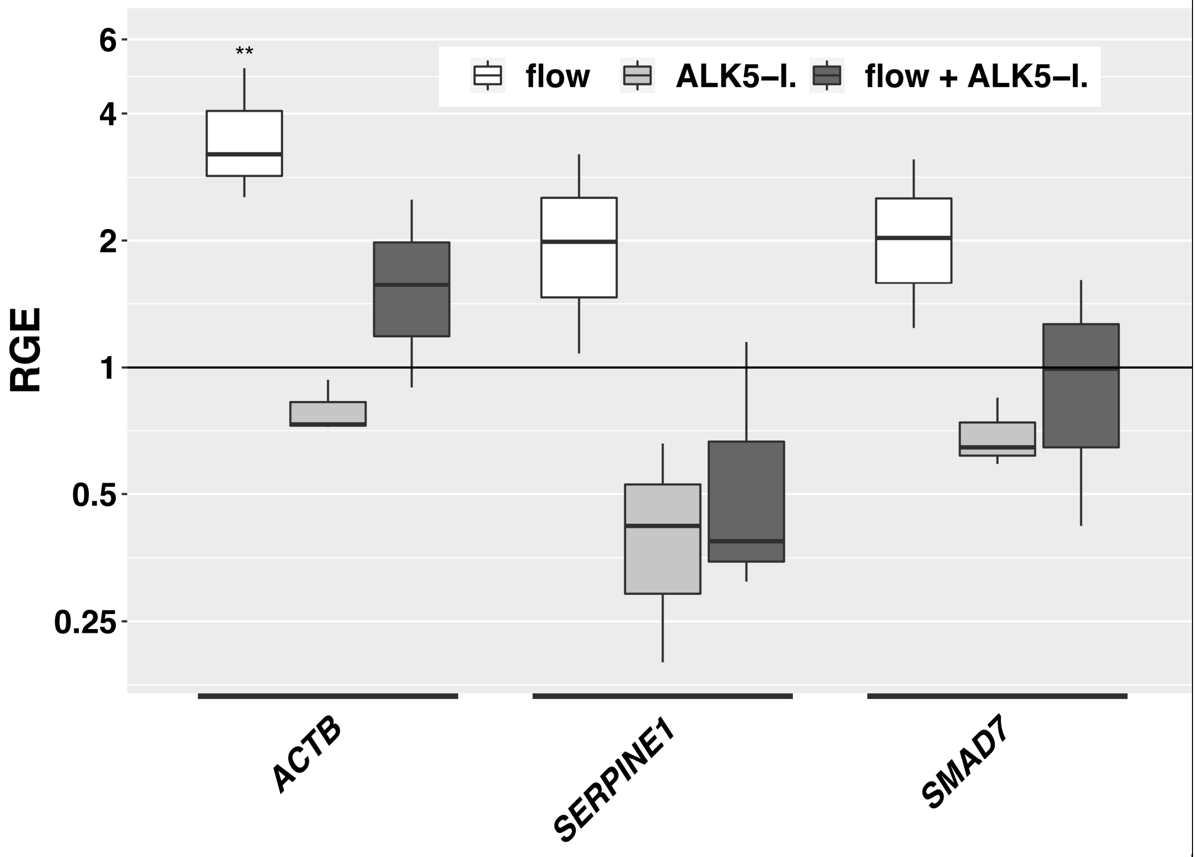

Fig. S4: Effects of ALK5-inhibition on the flow-induced changes in beta-actin and TGF-β-responsive genes SERPINE1 and SMAD7

Relative gene expression of actin-beta (*ACTB)*, Serpin Family E Member 1 (*SERPINE1)* and SMAD7 in the 3D model under flow (666 µl/h), static conditions

with ALK5-I. and flow conditions with ALK5-I. (0.1 µM, respectively) compared to static controls (72 h, n = 3). Asterisks indicate levels of significance according to Dunnett's t-test (*P<0.05, **P < 0.01, ***P<0.001).
